# Supplementary material for: Impact of Lifestyle Variables on Oral Diseases and Oral Health-Related Quality of Life in Children of Milan (Italy)
Source: Int J Environ Res Public Health. 2020 Sep 11;17(18):6612. doi: 10.3390/ijerph17186612 (PMC7559912; doi:10.3390/ijerph17186612)

Supplementary Material

Supplementary Table S1: numerosity, percentages chi-square test and chi-square proportion trend test results for disease.

Var_Name	Level	# No	% No	# Yes	% Yes	X^2P	X^2Ptrend	N.Missing
Sex	(Female)	53	34.6	100	65.4	0.127	0.099	7
	(Male)	60	44.1	76	55.9	0.127	0.099	7
Age	(6-7 Years)	34	28.3	86	71.7	0.004	0.000	3
	(8 Years)	30	40.5	44	59.5	0.004	0.000	3
	(9 Years)	22	51.2	21	48.8	0.004	0.000	3
	(10-11 Years)	30	53.6	26	46.4	0.004	0.000	3
Brest Feeding	(Yes)	16	30.8	36	69.2	0.187	0.140	0
	(No)	102	41.8	142	58.2	0.187	0.140	0
Tea Bottle	(No)	76	37.1	129	62.9	0.206	0.163	6
	(Yes)	39	45.9	46	54.1	0.206	0.163	6
Sugared Pacifier	(No)	104	38.7	165	61.3	0.564	0.418	6
	(Yes)	10	47.6	11	52.4	0.564	0.418	6
No Extra Meal Feeding	(Never)	53	43.1	70	56.9	0.749	0.294	3
	(Rarely)	13	43.3	17	56.7	0.749	0.294	3
	(Usually)	46	37.7	76	62.3	0.749	0.294	3
	(Always)	6	33.3	12	66.7	0.749	0.294	3
No Extra Fruit Juice	(Never + Rarely)	57	46.7	65	53.3	0.059	0.017	3
	(Usually)	40	37.7	66	62.3	0.059	0.017	3
	(Always)	19	29.2	46	70.8	0.059	0.017	3
No Junk Food	(Never)	40	41.2	57	58.8	0.774	0.785	2
	(Rarely)	12	44.4	15	55.6	0.774	0.785	2
	(Usually)	44	36.4	77	63.6	0.774	0.785	2
	(Always)	21	42.9	28	57.1	0.774	0.785	2
Toothbrush Before School	(Never + Rarely)	15	40.5	22	59.5	0.129	0.273	2
	(Usually)	21	53.8	18	46.2	0.129	0.273	2
	(Always)	80	36.7	138	63.3	0.129	0.273	2
Toothbrush Before Sleep	(Never + Rarely)	10	37.0	17	63.0	0.151	0.438	1

Var_Name	Level	# No	% No	# Yes	% Yes	X^2P	X^2Ptrend	N.Missing
	(Usually)	21	53.8	18	46.2	0.151	0.438	1
	(Always)	86	37.6	143	62.4	0.151	0.438	1
Fluoride Toothpaste	(Never + Rarely)	19	63.3	11	36.7	0.017	0.009	4
	(Usually)	34	41.0	49	59.0	0.017	0.009	4
	(Always)	64	35.8	115	64.2	0.017	0.009	4
Toothbrush Starting Age	(At Eruption)	22	31.4	48	68.6	0.353	0.181	12
	(1-2 Years)	36	39.1	56	60.9	0.353	0.181	12
	(3-4 Years)	45	45.0	55	55.0	0.353	0.181	12
	(> 4 Years)	8	36.4	14	63.6	0.353	0.181	12
Toothbrush Helping	(Always)	20	33.9	39	66.1	0.379	0.166	3
	(Sometimes)	61	38.9	96	61.1	0.379	0.166	3
	(Never)	35	45.5	42	54.5	0.379	0.166	3
Pediatrician Coaching	(No)	56	38.9	88	61.1	0.970	0.872	15
	(Yes)	52	38.0	85	62.0	0.970	0.872	15
Dentistry Coaching	(No)	9	15.8	48	84.2	0.000	0.000	10
	(Yes)	101	44.1	128	55.9	0.000	0.000	10
Dentistry Age	(0-5 Years)	56	48.3	60	51.7	0.399	0.629	56
	(6-7 Years)	39	39.8	59	60.2	0.399	0.629	56
	(8-11 Years)	13	50.0	13	50.0	0.399	0.629	56
Dentistry Cost Importance	(None + Low)	28	32.9	57	67.1	0.301	0.122	8
	(High)	47	39.2	73	60.8	0.301	0.122	8
	(Very High)	37	44.6	46	55.4	0.301	0.122	8
Lowercost Visit	(None)	24	36.9	41	63.1	0.156	0.142	9
	(Low)	16	26.7	44	73.3	0.156	0.142	9
	(High)	38	42.2	52	57.8	0.156	0.142	9
	(Very High)	32	44.4	40	55.6	0.156	0.142	9
Educational Qualification	(None – Middle School)	34	48.6	36	51.4	0.163	0.084	4
	(High School)	47	37.0	80	63.0	0.163	0.084	4
	(University)	33	34.7	62	65.3	0.163	0.084	4
ISEE	(<6500€)	37	46.8	42	53.2	0.010	0.007	70
	(6500€-12500€)	27	56.2	21	43.8	0.010	0.007	70

Var_Name	Level	# No	% No	# Yes	% Yes	X^2P	X^2Ptrend	N.Missing
	(12500€-27000€)	17	35.4	31	64.6	0.010	0.007	70
	(>=27000€)	13	25.5	38	74.5	0.010	0.007	70

Supplementary Table S2 numerosity, percentages chi-square test and chi-square proportion trend test results for oral health related quality of life (OrHQoL).

Var_Name	Level	# No	% No	# Yes	% Yes	X^2P	X^2Ptrend	N.Missing
Sex	(Female)	36	23.5	117	76.5	0.256	0.204	7
	(Male)	41	30.1	95	69.9	0.256	0.204	7
Age	(6-7 Years)	25	20.8	95	79.2	0.123	0.019	3
	(8 Years)	20	27.0	54	73.0	0.123	0.019	3
	(9 Years)	15	34.9	28	65.1	0.123	0.019	3
	(10-11 Years)	20	35.7	36	64.3	0.123	0.019	3
Brest Feeding	(Yes)	14	26.9	38	73.1	1.000	0.890	0
	(No)	68	27.9	176	72.1	1.000	0.890	0
Tea Bottle	(No)	47	22.9	158	77.1	0.005	0.003	6
	(Yes)	34	40.0	51	60.0	0.005	0.003	6
Sugared Pacifier	(No)	67	24.9	202	75.1	0.001	0.000	6
	(Yes)	13	61.9	8	38.1	0.001	0.000	6
No Extra Meal Feeding	(Never)	34	27.6	89	72.4	0.322	0.832	3
	(Rarely)	7	23.3	23	76.7	0.322	0.832	3
	(Usually)	38	31.1	84	68.9	0.322	0.832	3
	(Always)	2	11.1	16	88.9	0.322	0.832	3
No Extra Fruit Juice	(Never + Rarely)	43	35.2	79	64.8	0.008	0.002	3
	(Usually)	30	28.3	76	71.7	0.008	0.002	3
	(Always)	9	13.8	56	86.2	0.008	0.002	3
No Junk Food	(Never)	35	36.1	62	63.9	0.135	0.019	2
	(Rarely)	8	29.6	19	70.4	0.135	0.019	2
	(Usually)	29	24.0	92	76.0	0.135	0.019	2
	(Always)	10	20.4	39	79.6	0.135	0.019	2
Toothbrush Before School	(Never + Rarely)	13	35.1	24	64.9	0.023	0.033	2
	(Usually)	17	43.6	22	56.4	0.023	0.033	2
	(Always)	52	23.9	166	76.1	0.023	0.033	2

Var_Name	Level	# No	% No	# Yes	% Yes	X^2P	X^2Ptrend	N.Missing
	(Never + Rarely)	7	25.9	20	74.1	0.006	0.123	1
Toothbrush Before Sleep	(Usually)	19	48.7	20	51.3	0.006	0.123	1
	(Always)	55	24.0	174	76.0	0.006	0.123	1
	(Never + Rarely)	13	43.3	17	56.7	0.008	0.002	4
Fluoride Toothpaste	(Usually)	30	36.1	53	63.9	0.008	0.002	4
	(Always)	39	21.8	140	78.2	0.008	0.002	4
	(At Eruption)	12	17.1	58	82.9	0.153	0.026	12
Toothbrush Starting Age	(1-2 Years)	25	27.2	67	72.8	0.153	0.026	12
	(3-4 Years)	31	31.0	69	69.0	0.153	0.026	12
	(> 4 Years)	8	36.4	14	63.6	0.153	0.026	12
	(Always)	10	16.9	49	83.1	0.113	0.051	3
Toothbrush Helping	(Sometimes)	45	28.7	112	71.3	0.113	0.051	3
	(Never)	25	32.5	52	67.5	0.113	0.051	3
Pediatrician Coaching	(No)	38	26.4	106	73.6	1.000	0.907	15
	(Yes)	37	27.0	100	73.0	1.000	0.907	15
Dentistry Coaching	(No)	12	21.1	45	78.9	0.311	0.239	10
	(Yes)	66	28.8	163	71.2	0.311	0.239	10
	(0-5 Years)	39	33.6	77	66.4	0.080	0.561	56
Dentistry Age	(6-7 Years)	21	21.4	77	78.6	0.080	0.561	56
	(8-11 Years)	10	38.5	16	61.5	0.080	0.561	56
	(None + Low)	15	17.6	70	82.4	0.068	0.029	8
Dentistry Cost Importance	(High)	35	29.2	85	70.8	0.068	0.029	8
	(Very High)	27	32.5	56	67.5	0.068	0.029	8
	(None)	11	16.9	54	83.1	0.041	0.006	9
Lowercost Visit	(Low)	11	18.3	49	81.7	0.041	0.006	9
	(High)	27	30.0	63	70.0	0.041	0.006	9
	(Very High)	25	34.7	47	65.3	0.041	0.006	9
	(None – Middle School)	25	35.7	45	64.3	0.174	0.122	4
Educational Qualification	(High School)	31	24.4	96	75.6	0.174	0.122	4
	(University)	23	24.2	72	75.8	0.174	0.122	4
ISEE	(<6500€)	28	35.4	51	64.6	0.019	0.003	70

Var_Name	Level	# No	% No	# Yes	% Yes	X^2P	X^2Ptrend	N.Missing
	(6500€-12500€)	17	35.4	31	64.6	0.019	0.003	70
	(12500€-27000€)	10	20.8	38	79.2	0.019	0.003	70

Int. J. Environ. Res. Public Health **2020**, *17*, 6612; doi:10.3390/ijerph17186612 www.mdpi.com/journal/ijerph

Supplementary Figure S1: Logistic model analyses for both univariate and adjusted models for disease.

Disease (adjusted by sex and age)

Variable	Disease Yes/No	OR (CI 95%)	P. value	OR Adjusted (CI 95%)	P. value
Brest Feeding (Yes)	36 / 16				
(No)	142 / 102	1.616 (0.864 − 3.139)	0.142	1.523 (0.788 − 3.059)	0.222
Tea Bottle (No)	129 / 76				
(Yes)	46 / 39	1.439 (0.861 − 2.403)	0.164	1.32 (0.771 − 2.255)	0.309
Sugared Pacifier (No)	165 / 104				
(Yes)	11 / 10	1.442 (0.582 − 3.538)	0.42	1.247 (0.475 − 3.194)	0.646
No Extra Meal Feeding (Never)	70 / 53				
(Rarely)	17 / 13	1.01 (0.445 − 2.253)	0.981	1.178 (0.486 − 2.808)	0.712
(Usually)	76 / 46	0.799 (0.478 − 1.332)	0.391	0.77 (0.45 − 1.313)	0.338
(Always)	12 / 6	0.66 (0.218 − 1.817)	0.436	0.637 (0.202 − 1.833)	0.416
No Extra Fruit Juice (Never + Rarely)	65 / 57				
(Usually)	66 / 40	0.691 (0.405 − 1.172)	0.172	0.804 (0.449 − 1.436)	0.461
(Always)	46 / 19	0.471 (0.244 − 0.885)	0.022	0.515 (0.26 − 0.995)	0.051
No Junk Food (Never)	57 / 40				
(Rarely)	15 / 12	1.14 (0.476 − 2.692)	0.765	1.197 (0.47 − 2.995)	0.701
(Usually)	77 / 44	0.814 (0.47 − 1.41)	0.463	0.985 (0.555 − 1.756)	0.959
(Always)	28 / 21	1.069 (0.53 − 2.14)	0.851	1.263 (0.604 − 2.63)	0.533
Toothbrush Before School (Never + Rarely)	22 / 15				
(Usually)	18 / 21	1.711 (0.693 − 4.307)	0.247	1.574 (0.6 − 4.199)	0.358
(Always)	138 / 80	0.85 (0.42 − 1.761)	0.655	0.814 (0.389 − 1.745)	0.589
Toothbrush Before Sleep (Never + Rarely)	17 / 10				
(Usually)	18 / 21	1.983 (0.736 − 5.544)	0.181	1.741 (0.618 − 5.07)	0.299
(Always)	143 / 86	1.022 (0.454 − 2.412)	0.958	1.062 (0.458 − 2.583)	0.891
Fluoride Toothpaste (Never + Rarely)	11 / 19				
(Usually)	49 / 34	0.402 (0.165 − 0.938)	0.038	0.386 (0.152 − 0.943)	0.039
(Always)	115 / 64	0.322 (0.14 − 0.709)	0.006	0.36 (0.151 − 0.825)	0.017
Toothbrush Starting Age (At Eruption)	48 / 22				
(1−2 Years)	56 / 36	1.403 (0.732 − 2.726)	0.312	1.114 (0.557 − 2.24)	0.761
(3−4 Years)	55 / 45	1.785 (0.948 − 3.423)	0.076	1.474 (0.759 − 2.9)	0.255
(> 4 Years)	14 / 8	1.247 (0.442 − 3.364)	0.667	1.149 (0.393 − 3.206)	0.793
Toothbrush Helping (Always)	39 / 20				
(Sometimes)	96 / 61	1.239 (0.667 − 2.351)	0.503	1.016 (0.53 − 1.977)	0.961
(Never)	42 / 35	1.625 (0.811 − 3.311)	0.175	1.255 (0.599 − 2.652)	0.548
Pediatrician Coaching (No)	88 / 56				
(Yes)	85 / 52	0.961 (0.594 − 1.555)	0.872	0.976 (0.59 − 1.613)	0.924
Dentistry Coaching (No)	48 / 9				
(Yes)	128 / 101	4.208 (2.059 − 9.537)	0	4.107 (1.923 − 9.849)	0.001
Dentistry Age (0−5 Years)	60 / 56				
(6−7 Years)	59 / 39	0.708 (0.409 − 1.218)	0.214	0.736 (0.414 − 1.303)	0.294
(8−11 Years)	13 / 13	1.071 (0.454 − 2.527)	0.874	0.89 (0.363 − 2.173)	0.797
Dentistry Cost Importance (None + Low)	57 / 28				
(High)	73 / 47	1.311 (0.735 − 2.362)	0.362	1.197 (0.657 − 2.201)	0.559
(Very High)	46 / 37	1.637 (0.878 − 3.081)	0.123	1.352 (0.701 − 2.619)	0.368
Lowercost Visit (None)	41 / 24				
(Low)	44 / 16	0.621 (0.286 − 1.323)	0.221	0.618 (0.274 − 1.368)	0.239
(High)	52 / 38	1.248 (0.651 − 2.419)	0.507	1.262 (0.641 − 2.509)	0.503
(Very High)	40 / 32	1.367 (0.69 − 2.729)	0.372	1.311 (0.643 − 2.69)	0.457
Educational Qualification (None – Middle School)	36 / 34				
(High School)	80 / 47	0.622 (0.343 − 1.123)	0.115	0.524 (0.28 − 0.974)	0.042
(University)	62 / 33	0.564 (0.298 − 1.057)	0.075	0.471 (0.241 − 0.913)	0.026
ISEE (<6500€)	42 / 37				
(6500€−12500€)	21 / 27	1.459 (0.711 − 3.025)	0.304	1.766 (0.819 − 3.87)	0.15
(12500€−27000€)	31 / 17	0.622 (0.294 − 1.293)	0.208	0.726 (0.335 − 1.551)	0.411
(>=27000€)	38 / 13	0.388 (0.175 − 0.824)	0.016	0.433 (0.187 − 0.956)	0.043

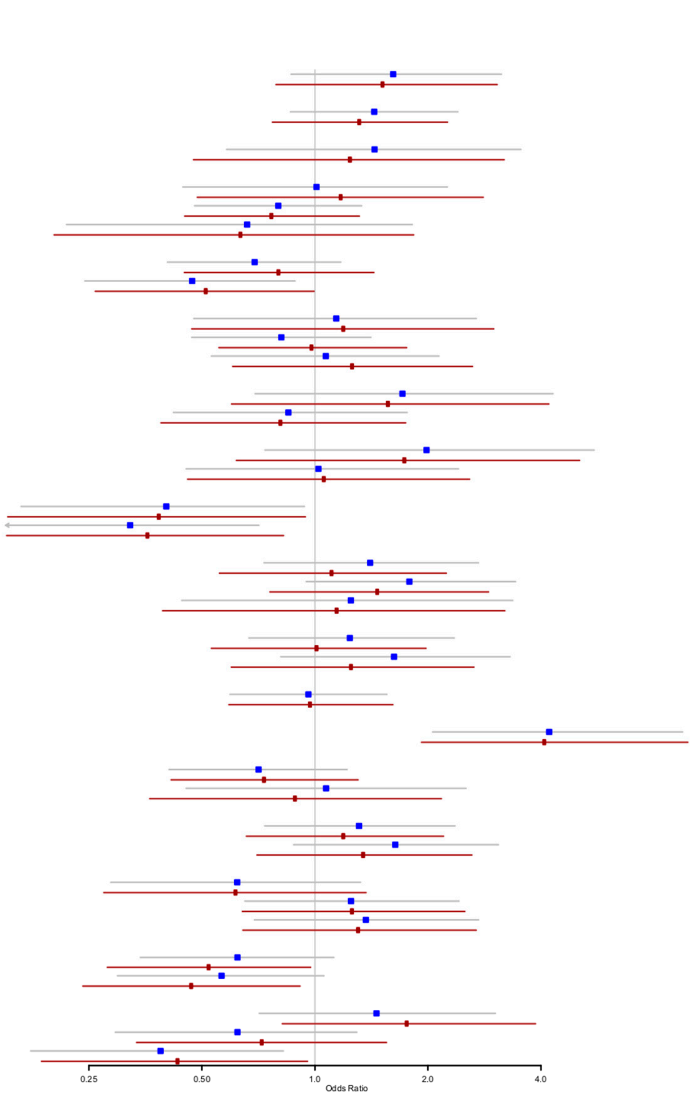

Int. J. Environ. Res. Public Health **2020**, *17*, 6612; doi:10.3390/ijerph17186612 www.mdpi.com/journal/ijerph

Supplementary Figure S2: Logistic model analyses for both univariate and adjusted models for oral health related quality of life (OrHQoL).

OrHQoL (adjusted by sex and age)

Univariate Multivariate

Variable	OrHQoL Yes/No	OR (CI 95%)	P. value	OR Adjusted (CI 95%)	P. value
Brest Feeding (Yes)	38 / 14				
(No)	176 / 68	1.049 (0.545 − 2.115)	0.89	0.967 (0.486 − 2.017)	0.925
Tea Bottle (No)	158 / 47				
(Yes)	51 / 34	2.241 (1.3 − 3.858)	0.004	2.165 (1.229 − 3.805)	0.007
Sugared Pacifier (No)	202 / 67				
(Yes)	8 / 13	4.899 (1.979 − 12.862)	0.001	4.72 (1.84 − 12.727)	0.001
No Extra Meal Feeding (Never)	89 / 34				
(Rarely)	23 / 7	0.797 (0.294 − 1.949)	0.633	0.814 (0.274 − 2.144)	0.69
(Usually)	84 / 38	1.184 (0.683 − 2.059)	0.547	1.165 (0.658 − 2.069)	0.601
(Always)	16 / 2	0.327 (0.05 − 1.233)	0.15	0.319 (0.048 − 1.234)	0.147
No Extra Fruit Juice (Never + Rarely)	79 / 43				
(Usually)	76 / 30	0.725 (0.411 − 1.269)	0.263	0.845 (0.456 − 1.556)	0.588
(Always)	56 / 9	0.295 (0.126 − 0.63)	0.003	0.332 (0.14 − 0.722)	0.008
No Junk Food (Never)	62 / 35				
(Rarely)	19 / 8	0.746 (0.283 − 1.831)	0.534	0.734 (0.259 − 1.906)	0.539
(Usually)	92 / 29	0.558 (0.308 − 1.003)	0.052	0.648 (0.351 − 1.191)	0.162
(Always)	39 / 10	0.454 (0.194 − 0.993)	0.056	0.48 (0.196 − 1.094)	0.092
Toothbrush Before School (Never + Rarely)	24 / 13				
(Usually)	22 / 17	1.427 (0.567 − 3.646)	0.452	1.33 (0.501 − 3.584)	0.567
(Always)	166 / 52	0.578 (0.278 − 1.244)	0.149	0.555 (0.259 − 1.235)	0.136
Toothbrush Before Sleep (Never + Rarely)	20 / 7				
(Usually)	20 / 19	2.714 (0.963 − 8.278)	0.066	2.44 (0.839 − 7.637)	0.11
(Always)	174 / 55	0.903 (0.377 − 2.404)	0.827	0.902 (0.369 − 2.448)	0.829
Fluoride Toothpaste (Never + Rarely)	17 / 13				
(Usually)	53 / 30	0.74 (0.317 − 1.751)	0.488	0.751 (0.31 − 1.852)	0.527
(Always)	140 / 39	0.364 (0.163 − 0.825)	0.014	0.419 (0.182 − 0.986)	0.042
Toothbrush Starting Age (At Eruption)	58 / 12				
(1−2 Years)	67 / 25	1.803 (0.847 − 4.016)	0.135	1.454 (0.659 − 3.326)	0.361
(3−4 Years)	69 / 31	2.171 (1.045 − 4.753)	0.043	1.815 (0.85 − 4.052)	0.132
(> 4 Years)	14 / 8	2.762 (0.93 − 8.053)	0.062	2.63 (0.868 − 7.822)	0.081
Toothbrush Helping (Always)	49 / 10				
(Sometimes)	112 / 45	1.969 (0.948 − 4.423)	0.082	1.685 (0.794 − 3.846)	0.191
(Never)	52 / 25	2.356 (1.051 − 5.611)	0.043	1.982 (0.857 − 4.836)	0.118
Pediatrician Coaching (No)	106 / 38				
(Yes)	100 / 37	1.032 (0.607 − 1.753)	0.907	1.064 (0.615 − 1.842)	0.824
Dentistry Coaching (No)	45 / 12				
(Yes)	163 / 66	1.518 (0.776 − 3.168)	0.241	1.427 (0.702 − 3.1)	0.344
Dentistry Age (0−5 Years)	77 / 39				
(6−7 Years)	77 / 21	0.538 (0.287 − 0.99)	0.049	0.532 (0.273 − 1.012)	0.058
(8−11 Years)	16 / 10	1.234 (0.499 − 2.942)	0.639	1.13 (0.438 − 2.815)	0.795
Dentistry Cost Importance (None + Low)	70 / 15				
(High)	85 / 35	1.922 (0.986 − 3.891)	0.061	1.805 (0.912 − 3.701)	0.096
(Very High)	56 / 27	2.25 (1.105 − 4.723)	0.028	1.925 (0.92 − 4.127)	0.086
Lowercost Visit (None)	54 / 11				
(Low)	49 / 11	1.102 (0.435 − 2.794)	0.836	1.102 (0.413 − 2.942)	0.845
(High)	63 / 27	2.104 (0.976 − 4.792)	0.065	2.29 (1.033 − 5.416)	0.048
(Very High)	47 / 25	2.611 (1.185 − 6.058)	0.02	2.699 (1.187 − 6.508)	0.021
Educational Qualification (None – Middle School)	45 / 25				
(High School)	96 / 31	0.581 (0.308 − 1.1)	0.094	0.488 (0.251 − 0.945)	0.033
(University)	72 / 23	0.575 (0.29 − 1.132)	0.11	0.469 (0.229 − 0.95)	0.036
ISEE (<6500€)	51 / 28				
(6500€−12500€)	31 / 17	0.999 (0.467 − 2.107)	0.998	1.07 (0.477 − 2.364)	0.868
(12500€−27000€)	38 / 10	0.479 (0.2 − 1.079)	0.084	0.516 (0.212 − 1.188)	0.13
(>=27000€)	44 / 7	0.29 (0.108 − 0.696)	0.008	0.276 (0.094 − 0.704)	0.011

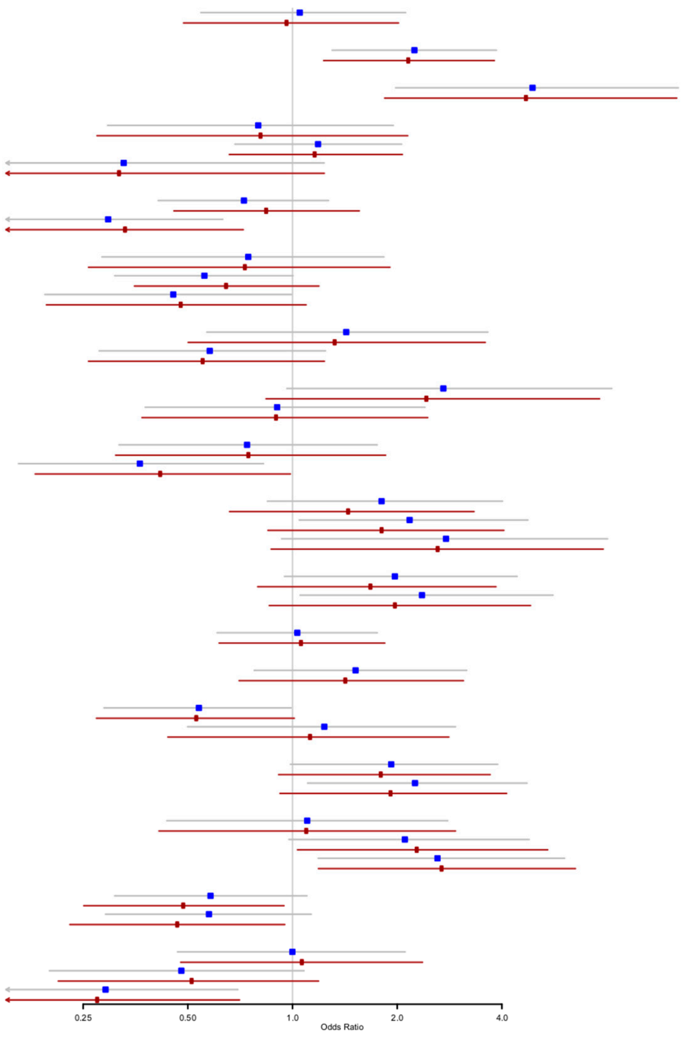

Supplement: Supplementary file 1 [file ijerph-17-06612-s001.pdf]
